# Case Report: Danon Disease: Six Family Members and Literature Review

**DOI:** 10.3389/fcvm.2022.842282

**Published:** 2022-05-20

**Authors:** Yuanyuan Wang, Meixue Jia, Yingjie Guo, Ting Zhang, Bin Ning

**Affiliations:** Department of Cardiology, People’s Hospital of Fuyang, Fuyang, China

**Keywords:** case report, Danon disease, lysosome-associated membrane 2, ventricular hypertrophy, pre-excitation, liver injury

## Abstract

Danon disease is a rare X-linked dominant genetic disorder that manifests with a clinical triad of cardiomyopathy, skeletal myopathy, and intellectual disability. It is caused by mutations in the lysosome-associated membrane 2 (LAMP2) gene. We report one case of Danon disease and his family members, characterized by ventricular pre-excitation, ventricular hypertrophy, abnormal muscle enzymes, and aberrant liver function. All the patients were confirmed to have Danon disease through genetic screening. Relevant literature was reviewed as a reference for the diagnosis and treatment of the disease.

## Introduction

Danon disease (DD; MIM#300257) refers to a rare lysosomal storage disease with various manifestations. Male patients display severe cardiomyopathy, skeletal myopathy, and developmental delay, while females show cardiac insufficiency and arrhythmia (1). Moreover, retinopathy in pigment epithelium, lens damage and unusual electroretinograms are also reported (2). DD was first described in 1981, in male members of a family who complained of limb weakness but were found with myocardial hypertrophy (3). The biopsy of skeletal muscle revealed vacuole bodies in the cytoplasm which contained autophagic material and glycogen. Nishino et al. (4) confirmed mutation of the lysosome-associated membrane 2 (LAMP2) gene (Xq24-q25) and the subsequent loss of LAMP-2 protein in these patients.

The pathology of DD includes vacuoles containing autophagic substances and the accumulation of massive glycogen in cardiomyocytes and skeletal muscle cells (1). Patients with DD display prominent ventricular hypertrophy and dilatation, with the former arising more in males. Female patients usually evince electrical conduction anomalies such as ventricular pre-excitation (5). Male patients with X-linked dominant inheritance pass the tendency to daughters, but not sons. Therefore, more females than males theoretically suffer from DD, although for the atypical symptoms, most cases are not identified in females. Mild cardiac disorders will develop swiftly into end-stage heart failure in the third decade of life unless the patient receives heart transplantation (6).

The present study reports one case of DD and his family members within three generations (see Supplementary Figure 1 for the comprehensive pedigree). To provide a reference for clinical practice, we searched more than 15 years of medical history and relevant literature in PubMed to review the manifestations, causes, diagnosis, therapy, and prognosis of DD.

## Methods

The six patients were collected from 2003 to 2021 at Fuyang People’s Hospital in the province of Anhui, China. All the medicines, medical procedures, and mechanotherapy conformed to the recommendation of the Chinese Guidelines for Diagnosis and Treatment of Heart Failure (2018) and Atrial Fibrillation: Current Understanding and Treatment Recommendations—2018 (7, 8).

## Report on the Case and Family Members

### Case (III4)

**FIGURE 1:**
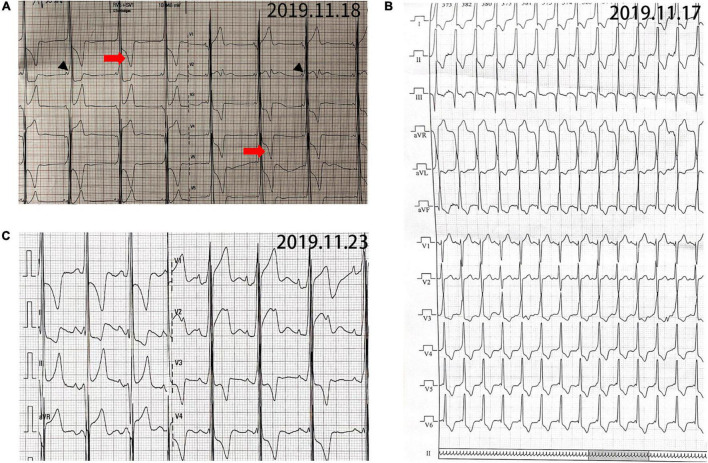
Electrocardiogram (ECG) of patient 1. **(A)** ECG before operation showing sinus rhythm and 74 ms of PR interval. Delta-wave (black arrowhead) was present in II and III leads. RV_5_ > 2.5 mV and RV_5_ + SV_*l*_ > 4.0 mV in precordial leads. RI > 1.5 mv, RaVL > 1.2 mv, RaVF > 2.0 mv with secondary ST-T changes (red arrow). **(B)** Narrow QRS tachycardia indicating atrioventricular reentrant tachycardia (AVRT). **(C)** ECG after operation showing sinus rhythm and 144 ms of PR interval. No δwave presented, but the hypertrophic remodeling of left ventricle still existed.

**FIGURE 2:**
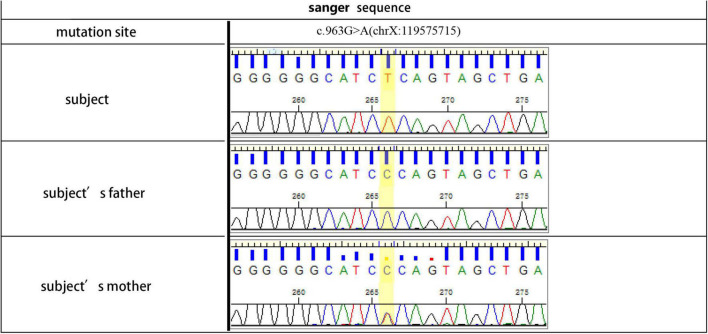
Gene sequence of the III4 (Pt 1, subject), II3 (subject’s father) and II4 (Pt 2, subject’s mother).

The proband (Pt 1) was a male aged 16 years who was admitted to hospital for palpitation. The electrocardiogram (ECG) showed overt ventricular pre-excitation with 74 ms of PR interval, > 2.5 mV of R_*V5*_, > 4.0 mV of R_*V5*_ + S_*Vl*_, > 1.5 mV of R_*I*_, > 1.2 mV of R_*aVL*_, and > 2.0 mV of R_*aVF*_, which suggested a hypertrophic remodeling of the left ventricle. Secondary ST-T changes were present in all leads and Delta-waves were detected in II and III leads. The narrow QRS tachycardia indicated atrioventricular reentrant tachycardia (Figures 1A,B). The serum concentrations of creatine kinase (1129.6 U/L), alanine transferase, and aspartate aminotransferase were higher than normal. Echocardiography detected hypertrophy in the ventricular septum and left ventricular wall (Supplementary Figure 2). A diffused thickness of the left ventricle and multiple patchy delayed enhancements were observed on the magnetic resonance imaging (MRI, Supplementary Figure 3). Pt 1 received radiofrequency ablation (RFA) of multiple atrioventricular accessories. After the treatment, the pre-excitations in the ECG disappeared and the PR interval was restored, but the hypertrophic remodeling of the left ventricle remained (Figure 1C). The patient has been taking metoprolol tartrate (25 mg/d) and benazepril (5 mg/d) orally. Motor function and muscle strength are normal. A genetic test revealed a hemizygous mutation in the LAMP2 gene (LAMP2: c.963G > A: Figure 2). Throughout the treatment period, the patient showed a quite good compliance and tolerance and was satisfied with the curative effect. During the subsequent 2 years of follow-up, echocardiography still indicated a hypertrophic wall of the left ventricle, and the serum creatine kinase remained abnormal (1,432 U/L, 2 months after the treatment). The patient reported average muscular strength, but low performance in primary school (not verified by professional tests). There was no indication of heart failure in the patient.

### Family Member 1 (II4)

**FIGURE 3:**
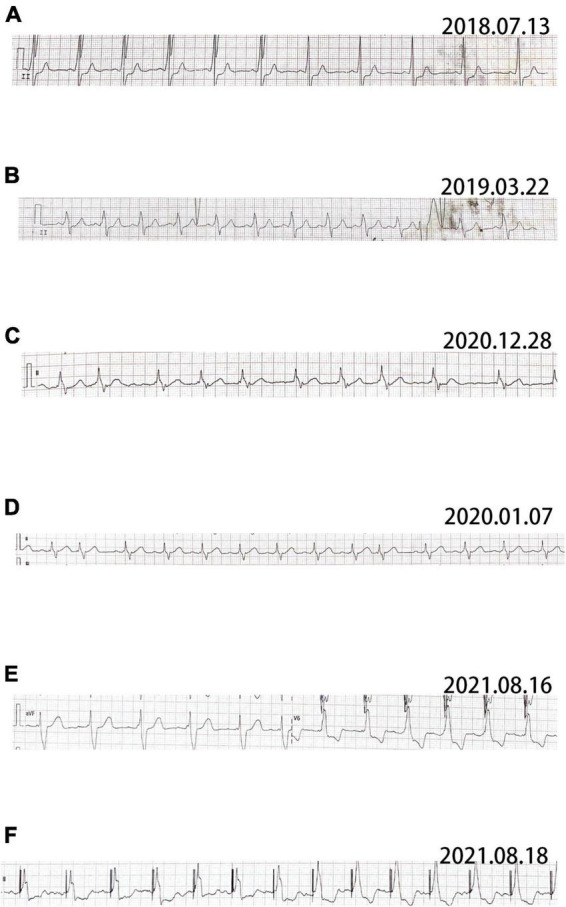
ECG of patient 2. **(A)** The earliest ECG of patient showing sinus rhythm and complete left bundle branch block (CLBBB) with the width of QRS wave of 150 ms. **(B)** Sinus rhythm, CLBBB and frequent ventricular premature beat. **(C)** Atrial fibrillation and CLBBB. **(D)** Recovered sinus rhythm after radiofrequency ablation. **(E)** The pre-operative ECG showing sinus rhythm, CLBBB and 220 ms of QRS wave width. **(F)** Post-operative ECG showing sinus rhythm and 190 ms of QRS wave width.

Pt 1’s mother (Pt 2), aged 38 years, was repeatedly admitted to hospital due to palpitation, chest tightness, short of breath or low endurance to activity. Atrioventricular reentrant tachycardia and hypertrophic cardiomyopathy (HCM) was diagnosed. The electrophysiological examination suggested a concealed accessory pathway in the right anterior interventricular septum. Echocardiography and MRI revealed a thick interventricular septum and posterior wall in the left ventricle (Table 1 and Supplementary Figures 4, 5). From 2003 to 2021, the patient’s condition aggravated bit by bit, successively developing dilated cardiomyopathy, complete left bundle branch block (CLBBB), ventricular premature beats with a QRS wave width of 150 ms (Figures 3A,B), and paroxysmal atrial fibrillation (Figure 3C). The cardiac function attenuated to New York Heart Association (NYHA) functional classification III. Transesophageal echoaortography revealed left atrial appendage thrombosis. After 3 months of regular anticoagulation therapy, the patient received RFA (Supplementary Figure 6), which recovered sinus rhythm (Figure 3D) and improved the cardiac function to NYHA II. However, her condition worsened again soon after the procedure, with an enlarged heart, moderate pulmonary hypertension, right heart failure, and liver failure. The cardiac function was at NYHA III-IV, which improved to NYHA II after medication treatment, but this improvement also lasted for a short time. The patient was soon barely able to get out of bed and the ECG showed sinus tachycardia and CLBBB with a QRS wave width of 220 ms (Figure 3E). Multiple organ failure appeared. Standard medication treatment failed to alleviate the situation and the blood pressure of the patient fluctuated at 75–85/50–60 mmHg. Therefore, a cardiac resynchronization therapy defibrillator (CRT-D) was implanted (Supplementary Figure 7). The post-operative ECG showed sinus rhythm and biventricular pacing with 190 ms of QRS wave width (Figure 3F). The blood pressure stayed above 95/65 mmHg, with the clinical symptoms overtly improved. Electromyogram (EMG) revealed no abnormality all the way. The patient also received funduscopy examination which found normal cup to disk ratio and no hypopigmentation. The medication of the patient included oral metoprolol (23.75 mg/d), spironolactone (20 mg/d), furosemide (20 mg/d) and benazepril (5 mg/d), which was later replaced by valsartan (50 mg/d). At the same time, amiodarone (0.2 g/d) and rivaroxaban (15 mg/d) were given to manage arrhythmia and hypercoagulability, respectively. During the treatment, the patient consistently complained of fatigue, and she remains under follow-up as of this writing. The genetic testing unveiled a similar mutation in LAMP2, as in Pt 1 (Figure 2).

**TABLE 1:** Echocardiograph indexes of Pt 2.

	Echocardiograph indexes, mm							
	LA	LV	RA	RV	IVS	LVPW	EF (%)	Note
2003/12/22	31.2	42.4	Normal	Normal	13.5	PWT13.5, AWT14.0, SWT13.8	60	
2012/5/23	36	46.2	Normal	Normal	10.4	PWT10, SWT11.9	59	Mild mitral and tricuspid regurgitation
2014/8/17	32	46	31	17	15	13	50	
2015/3/8	33	40	30	18	14	12	52	
2015/11/21	33	45	30	16	13	13	56	Mild mitral and tricuspid regurgitation
2017/11/21	34	46	30	16	11	10	59	Mild mitral regurgitation
2019/3/22	36	45	34	20	10	10	51	Mild tricuspid regurgitation; mild pulmonary artery hypertension
2020/6/28	36	56	34	22	7	8	41	Mild mitral regurgitation
2020/9/25	40	59	Normal	Normal	13	11	31	Mild mitral regurgitation; Left atrial appendage thrombosis
2021/1/4	42	57	Normal	Normal	13	11	24	Mild mitral and tricuspid regurgitation; Left auricular hypercoagulability; mild pulmonary artery hypertension
2021/6/29	40	55	42	38	10	8	34	Mild mitral and tricuspid regurgitation; Moderate pulmonary artery hypertension (55 mmHg)
2021/8/16	42	59	Normal	Normal	13	11	22	Mild mitral regurgitation; Moderate tricuspid regurgitation; mild pulmonary artery hypertension (41 mmHg)

*EF, ejection fraction; IVS, interventricular septum thickness; LA, left atrium; LV, left ventricle; LWPW, left ventricular posterior wall thickness; RA, right atrium; RV, right ventricle.*

### Family Member 2 (II2)

Pt 3 was the elder sister of Pt 2, aged 40 years, and was hospitalized twice for chest tightness. The coronary angiography showed coronary atherosclerosis and coronary myocardial bridge. The ECG indicated atrial premature contractions, ventricular premature contractions, and poor R wave progression. The echocardiography showed enlargement of both atriums, but normal ventricular wall thickness (Supplementary Figure 8). Heart MRI showed diffuse late gadolinium enhancement in the subendocardium and midmyocardium of the left ventricular wall (Supplementary Figure 9). The EMG was normal for this patient. The medication consisted of oral metoprolol (50 mg/d), furosemide (20 mg/d), spironolactone (20 mg/d), warfarin (2.5 mg/d) and valsartan (50 mg/d). The genetic test revealed the similar mutation of LAMP2 as in Pt 1. At present, Pt 3 performs normal daily activities competently. The most recent evaluation showed NYHA I cardiac function.

### Family Member 3 (II6)

Pt 4 was the younger sister of Pt 2, without a medical record. The echocardiography found no anomaly. ECG showed sinus rhythm and aberrant Q wave on the I and aVL leads. The genetic test revealed that she and her two sons held the similar mutation of LAMP2 as in Pt 1. During the follow-up, the patient reported a normal life.

### Family Member 4 (III5)

Pt 5, aged 8 years, was one of the sons of Pt 4. The genetic test indicated the mutation of LAMP2 as in Pt 1. No clinical manifestation was detected except higher serum concentrations of creatine kinase, lactate dehydrogenase, α-hydroxybutyrate dehydrogenase, alanine transferase, and aspartate aminotransferase. The ECG and echocardiograph suggested hypertrophic ventricles. The follow-up found no abnormity in the patient.

Family member 5Pt 6, aged 2 years, was another son of Pt 4. The genetic test revealed the similar mutation in LAMP2 as for Pt. 1, and the echocardiograph showed a thickened ventricular wall. The patient remained healthy during the follow-up.

## Discussion

Limited studies have shown a 4–6% rate of DD in pediatric patients with HCM, 0.7–4% in adults with HCM, 6–8% in adults with symmetric HCM, 17–30% in LV hypertrophy with pre-excitation, and 33% in HCM with vacuolar cardiomyopathy (9–11). All patients with DD develop cardiomyopathy, with males more prone to HCM, and females to HCM or dilated cardiomyopathy. In addition, most female patients present with mild symptoms and later onset compared with males (6). Electrocardiographic anomaly exists in 86–100% of male patients with DD (12), which is in accord with the present results that 5 patients of 6 evinced significant ECG disorders. Therefore, HCM accompanied with pre-excitation syndrome in a young male strongly implies DD. The underlying mechanisms remain debated, but may be associated with myocardial hypertrophy, defective autophagy, or microscopic atrioventricular connection (13).

Atrioventricular bundle passage could be a sign of DD, and 35–50% of patients with DD suffering from heart block are in need of pacemaker implantation (1). Moreover, frequent atrial fibrillation (AF), flutter, and life-threatening ventricular arrhythmias appear in 60% of DD cases (14). AF may aggravate heart failure, so when confirmed, should be watched for closely. RFA for cases of DD may not be successful due to resistance of diffuse fibrosis (1). In the present study, both Pt1 and Pt2 benefited from RFA, indicating that the fibrosis may not have been severe.

The left ventricular hypertrophy develops into dilated cardiomyopathy (DCM) in 10–33% of DD cases (6). This condition tends to quickly worsen to heart failure (15). Cardiac MRI, especially the T1 mapping and extracellular volume measurement, helps the diagnosis and prognostic assessment of DD (15, 16). Myocardial fibrosis signs in MRI determine the necessity of implantable cardioverter-defibrillator installation or heart transplantation (16).

Cardiac embolic stroke and ischemic thromboembolic stroke usually arise in DD (17) indicating a poor prognosis, so the anticoagulation therapy should be arranged when representative signs appear in MRI. The situation of Pt 2 in our study was relatively complicated with the onset of heart failure and left atrial appendage thrombosis. However, the anticoagulation treatment still apparently improved the condition and allowed the patient the opportunity to get RFA.

Skeletal muscle lesions are present in 80–100% of male patients with DD, which in general is mild, but turns severe occasionally and is always accompanied with 3-to-35 times higher serum creatine kinase activity (18). Females are relatively free from skeletal muscle injury and aberrant creatine kinase (to twice higher than normal). Moreover, hepatomegaly, hepatopathy, and elevated liver enzymes in childhood appear frequently in DD (1). In our results, the motor function and muscle strength of the six patents were normal, but the serum creatine kinase content become higher in Pt1 and Pt5 indicating the possible damage of skeletal muscles.

At least 110 types of mutations have been reported in the LAMP2 gene, of which c.926G > A is the commonest. Most mutations were non-functional (i.e., insignificant or a frameshift lack/insert) or splicing. Missense mutation, recombination, or synonymous substitution are rare (6). It has been reported that the eight mutations of LAMP2 accounts for the specific manifestations of DD including microdeletions, insertions, non-sense point mutations, intronic point mutations and 10-bp deletion (19). In the present study, the c.963G > A mutation was a novel discovery.

As for the treatment of DD, the ultimate strategy should be heart transplantation, especially for the female carriers (18, 20). Before the operation, a left ventricular assist device should be applied at a right time as it is crucial for maintaining the residual cardiac capacity. To avoid sudden death, cardioverter defibrillator is also a useful treatment, for which the subcutaneous is more impressive than intravenous implantation in ending ventricular tachycardia (21). With the growing utilization of molecular genetic tests in cardiomyopathy of unknown origin, more DD cases will be recognized. However, for now, genetic and protein therapies remain unavailable (22, 23).

In the present study, the DD arose earlier in the males. As seen in Pt 6, the onset age was just 2 years. All the males showed typical clinical manifestations such as LVH, and one developed Wolff-Parkinson-White syndrome. Thus, in a youth with HCM and ventricular pre-excitation, DD should be strongly considered. Liver dysfunction and skeletal muscular impairment were also more common in males in the present study; the females showed diversified cardiac manifestations, and two developed heart failure during follow-up. The definitive gender difference implies that DD occurs later in females than in males, at the age of about 30 years. The two female patients in our study displayed atrial arrhythmias at the beginning, which exacerbated the heart failure significantly. The gender-associated difference in clinical manifestations may be due to estrogen, which promotes autophagy and a lysosome pathway in multiple cells (24).

Regarding diagnosis, a family genetic screen and carrier deduction based on a genetic map should be conducted. Moreover, the cardiac MRI of the three patients in the present study identified delayed enhancement in the myocardium and high T1-mapping, which is valuable for the differential diagnosis of patients with HCM.

If clinical manifestations are not present, a close follow-up is necessary. With the advent of Wolff-Parkinson-White syndrome or atrial arrhythmias, medication or RFA should be considered. Malignant ventricular arrhythmia demands the installation of an implantable cardioverter-defibrillator. If the condition has developed into heart failure, active medication treatment should be effective. For patients who are insensitive to medication, an active CRT-D implantation is suggested.

It has been mentioned that some DD cases develop abnormalities in cerebral vessels (25), which, however, was not examined in our case series. This is a limitation of the study.

Overall, the auxiliary device implantation at an early stage would improve heart function or defer the exacerbation of cardiac performance in patients of DD. However, all these treatments are only palliative or delay the deterioration of the condition. Without heart transplantation or future gene therapy, most patients will die within the ages of 40–50 years. Unfortunately, although we have recommended patient 2 to make an appointment for HTx, they refused to follow the suggestion for economic reasons or the lack of understanding on severity of the disease.

## Data Availability Statement

The original contributions presented in the study are included in the article/Supplementary Material, further inquiries can be directed to the corresponding author/s.

## Ethics Statement

The studies involving human participants were reviewed and approved by the Ethics Committee of the People’s Hospital of Fuyang. Written informed consent to participate in this study was provided by the participants’ legal guardian/next of kin. Written informed consent was obtained from the individual(s) for the publication of any potentially identifiable images or data included in this article.

## Author Contributions

BN: conceptualization, supervision, and writing—review and editing. YW: data curation and writing—original draft. MJ: data curation and formal analysis. YG: formal analysis and supervision. TZ: data curation. All authors read and approved the final manuscript.

## Conflict of Interest

The authors declare that the research was conducted in the absence of any commercial or financial relationships that could be construed as a potential conflict of interest.

## Publisher’s Note

All claims expressed in this article are solely those of the authors and do not necessarily represent those of their affiliated organizations, or those of the publisher, the editors and the reviewers. Any product that may be evaluated in this article, or claim that may be made by its manufacturer, is not guaranteed or endorsed by the publisher.
