# Supplementary material for: Case Report: Danon Disease: Six Family Members and Literature Review
Source: Front Cardiovasc Med. 2022 May 20;9:842282. doi: 10.3389/fcvm.2022.842282 (PMC9163303; doi:10.3389/fcvm.2022.842282)
Supplement: Supplementary file 1 [file Data_Sheet_1.docx]

Supplementary Material


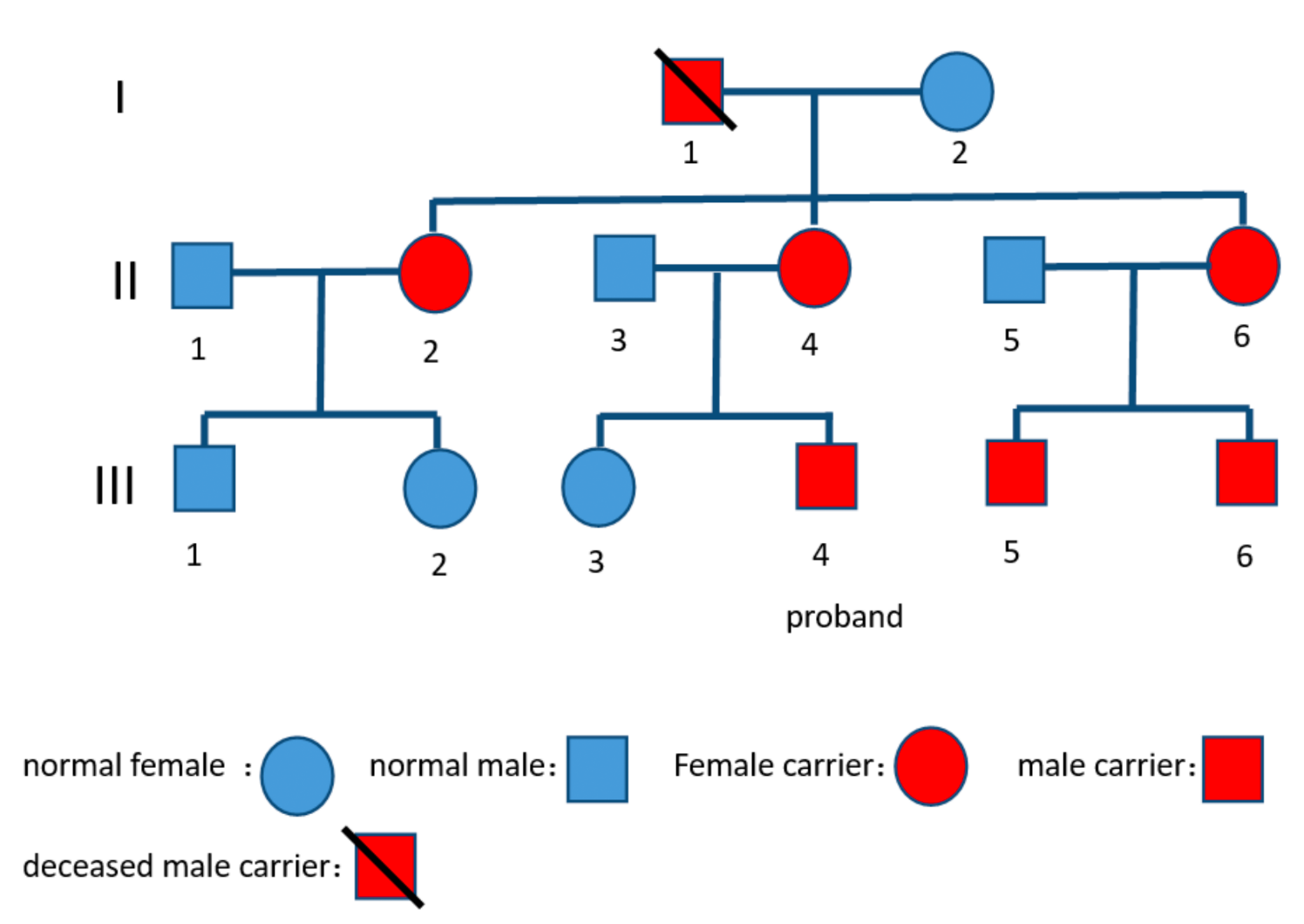


**Supplemental Figure 1.** Family Pedigree of the patients


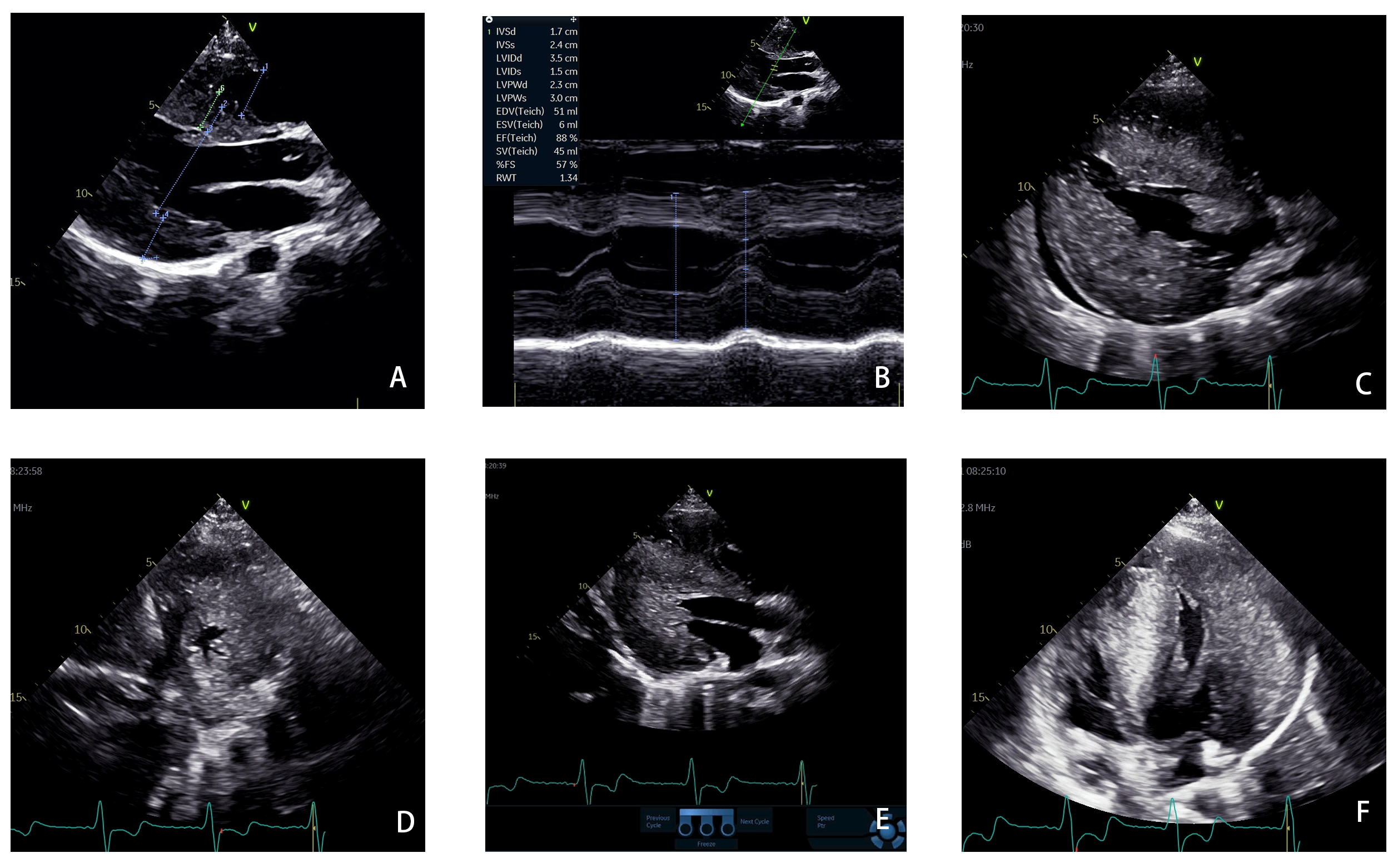


**Supplemental Figure 2.** Long axis and short axis sections of left ventricle in echocardiograph of patient 1, indicating the thickening of interventricular septum and left ventricular wall. The maximum thickness attained 36 mm with the thickest section of right ventricle of 12 mm (pentagram). The white arrow denoted a small quantity of pericardial effusion.


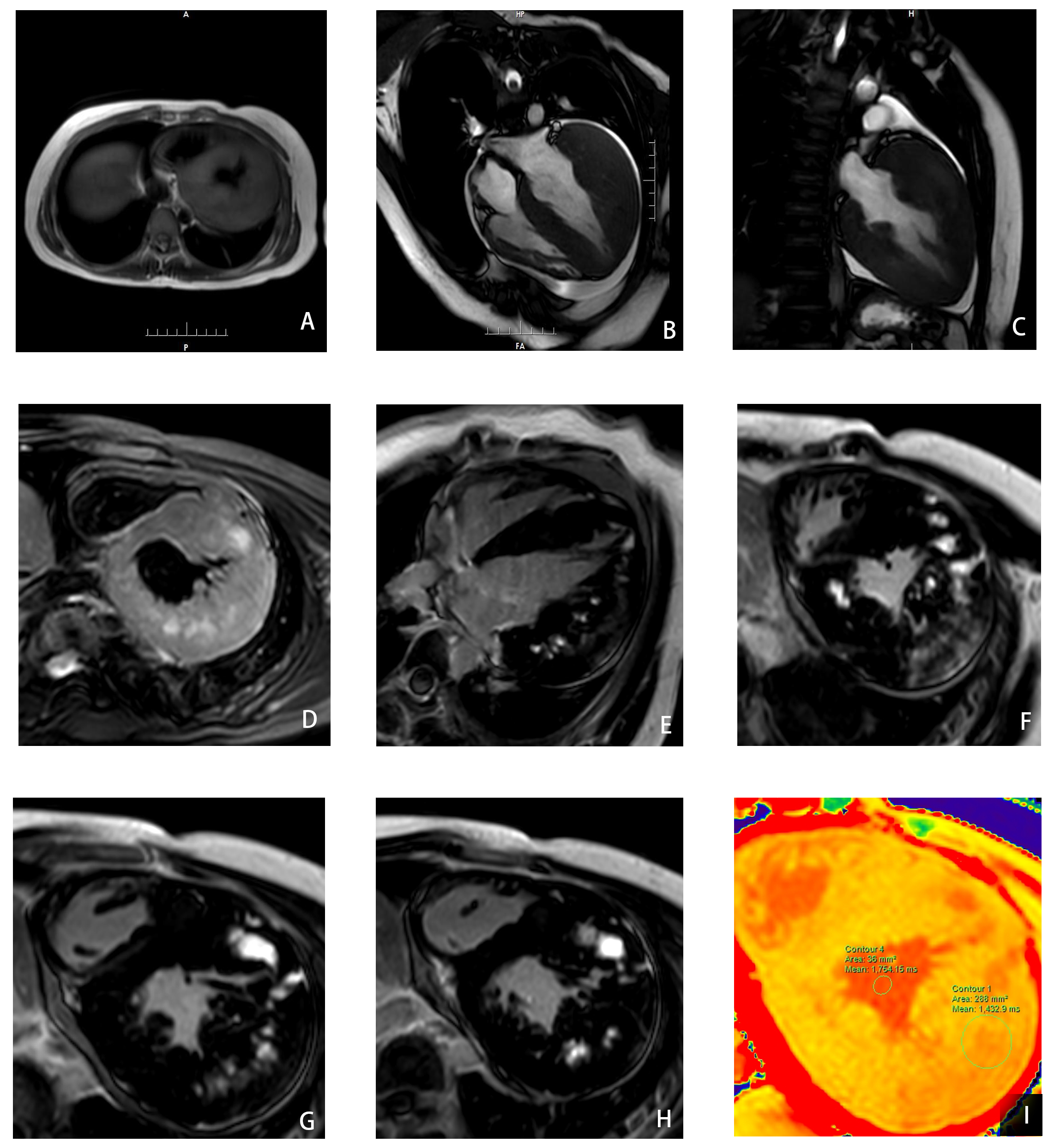


**Supplemental Figure 3.** Magnetic resonance imaging (MRI) of the myocardium in patient 1. A, B and C showed a thickened left ventricular wall with 30 mm of maximal end-diastolic thickness, 10 mm of right ventricle wall thickness, 43 mm of left ventricular end diastolic diameter (LVEDD), 65% of left ventricular ejection fraction and pericardial effusion. Patchy T2W1 high signal appeared in medial myocardium of left ventricle (D). Patchy late gadolinium enhancement (LGE) appeared in left ventricular free wall, but not septal wall which were increased gradually from middle to apex of left ventricle (E-I). I, T1 mapping at 1432.9 ms.


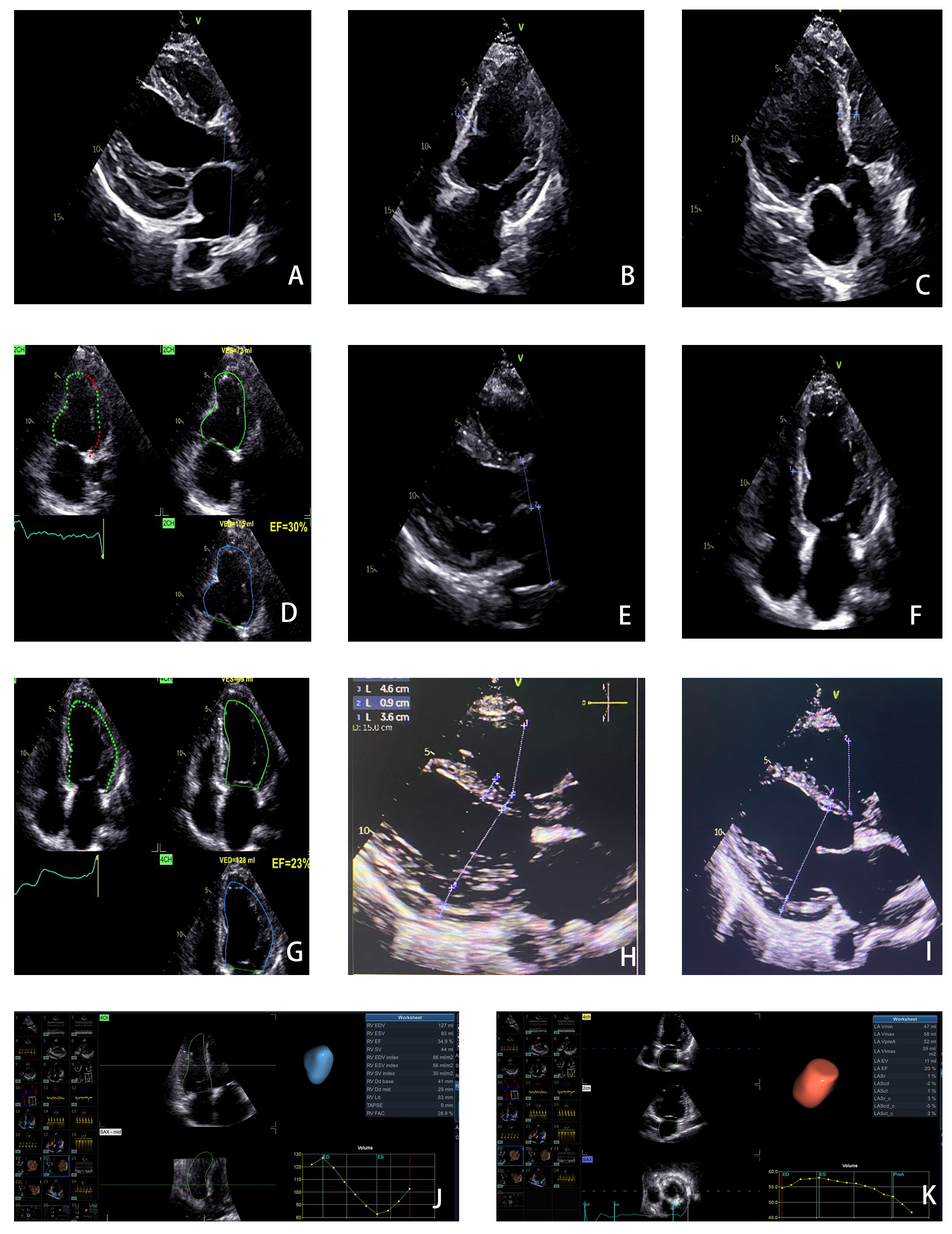


**Supplemental Figure 4.** Echocardiography of patient 2. A, B, C, L (September, 2020), 40 mm of left atrial wall thickness, 59 mm of left ventricular thickness, 31% of EF, 13 mm of interventricular septum thickness and 11 mm of left ventricular posterior wall thickness. H-K, 40 mm of left atrial wall thickness, 55 mm of left ventricular thickness, 42 mm of right atrial wall thickness, 38 mm of right ventricular thickness, 39ml/m2 of LAVImax, 20% of LAEF, 0.84 of RAAR, 0.35 of RVEF, 0.34 of LVEF and the estimated pulmonary arterial systolic pressure from TR of 55 mmHg.


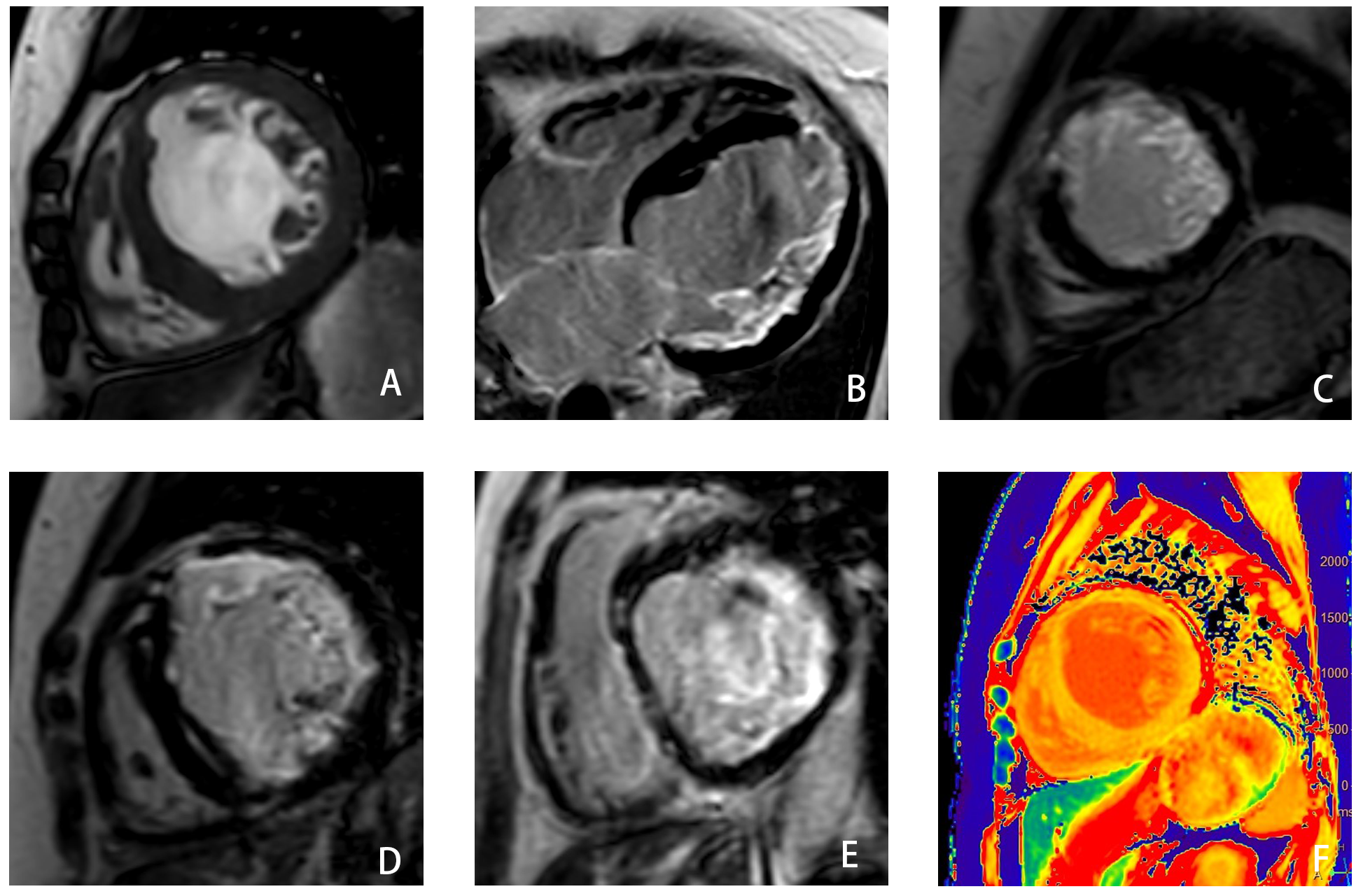


**Supplemental Figure 5.** Cardiac MRI of patient 2. A, enlarged left ventricular volume, 60 mm of left ventricular end diastolic diameter, slightly thickened left ventricular wall, 15 mm of maximal end-diastolic thickness, 24% of left ventricle EF. B-E, PSIR delayed enhanced sequence on left ventricular long axis section, base section, central section and apex short axis section showing diffuse subendocardial LGE in left ventricular free wall gradually increased from base to apex. Streaky LGE presented in medium myocardium of interventricular septum. F, T1 mapping at 1382.9ms.

**
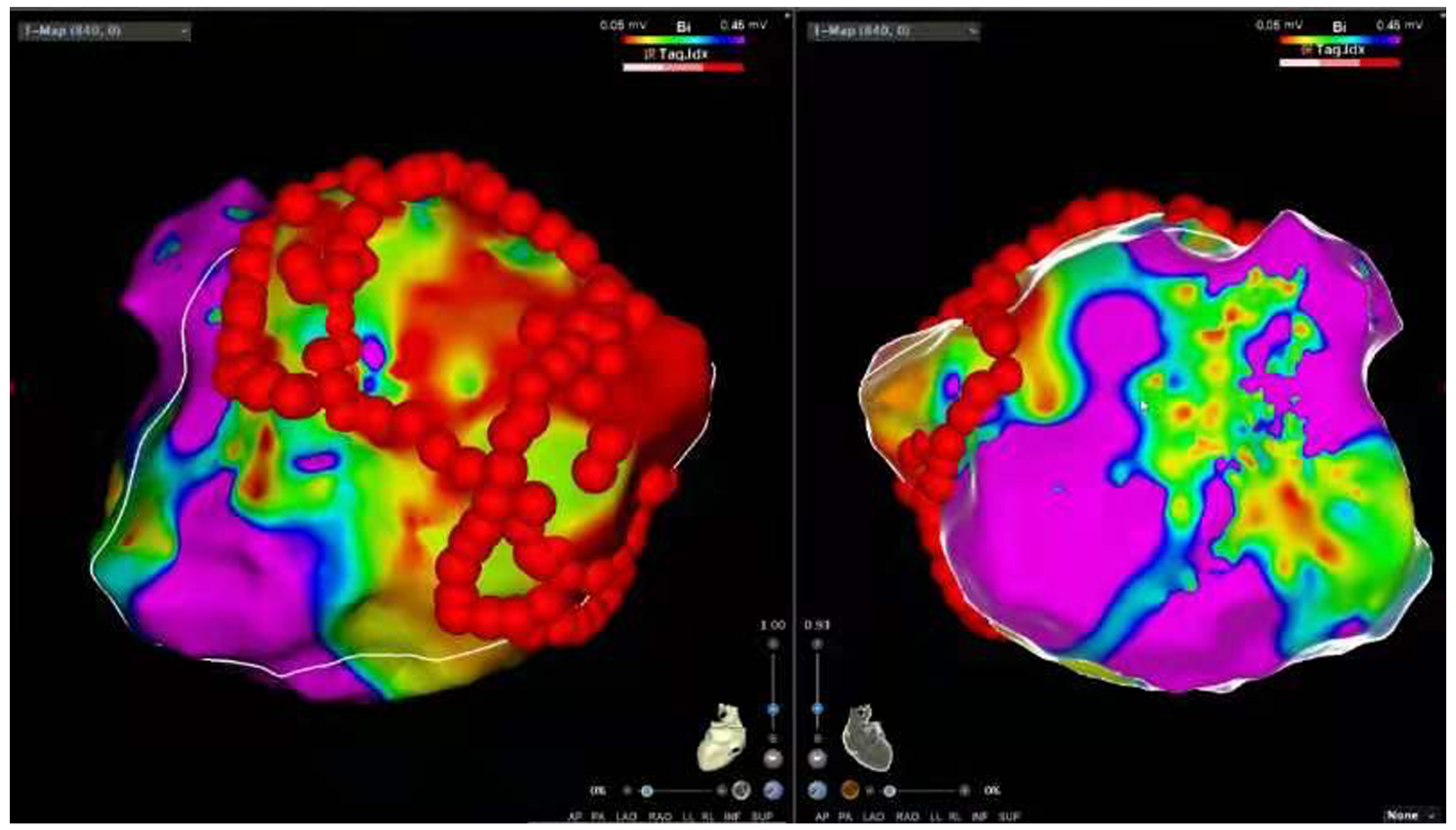
**

**Supplemental Figure 6.** Radiofrequency ablation (CPV+BOX) for treating atrial fibrillation of patient 2. The matrix measure indicated a poor atrium condition.

**
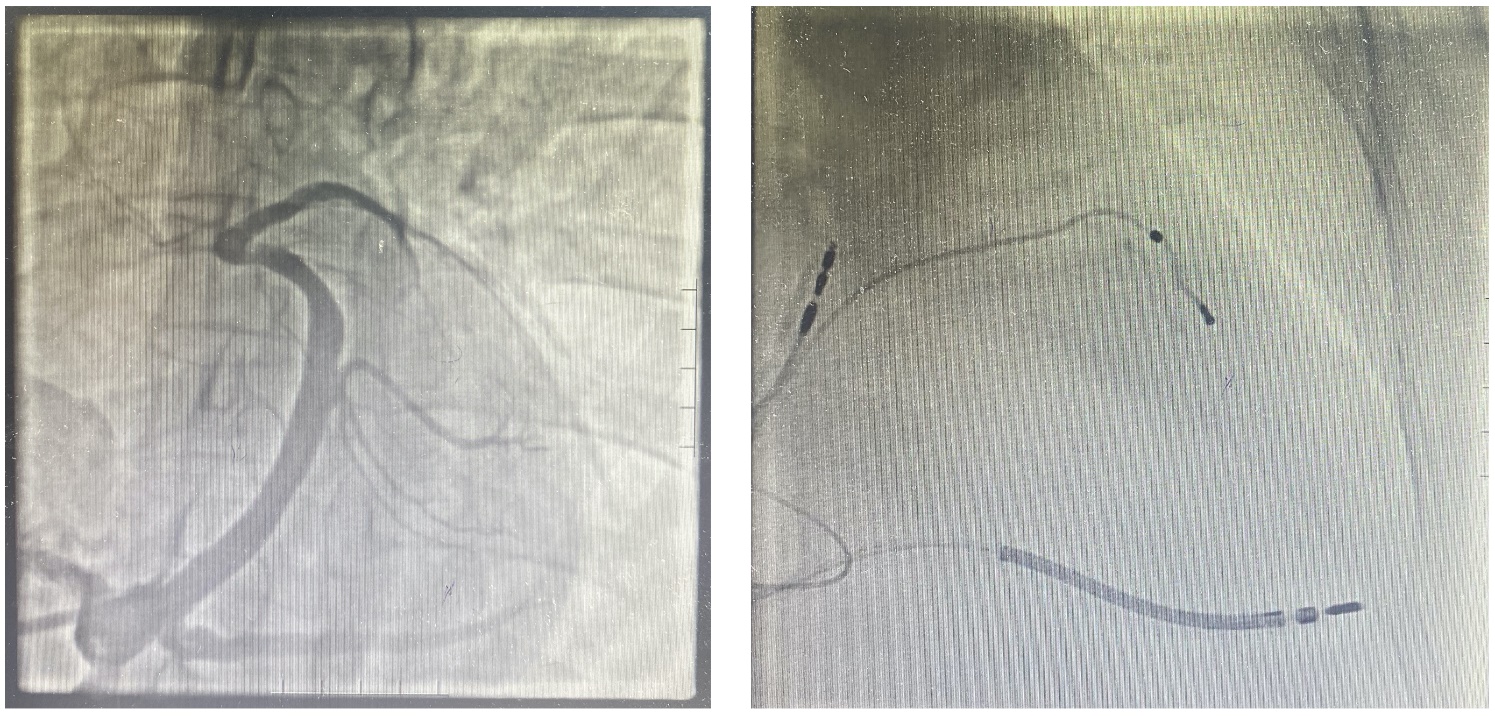
**

**Supplemental Figure 7.** Coronary venography of patient 2. A, Frontal film. B, Anteroposterior film showing the placement of left ventricular electrode wire on lateral vein, right ventricular electrode wire on inferior ventricular septum, right atrial electrode wire on right appendage.


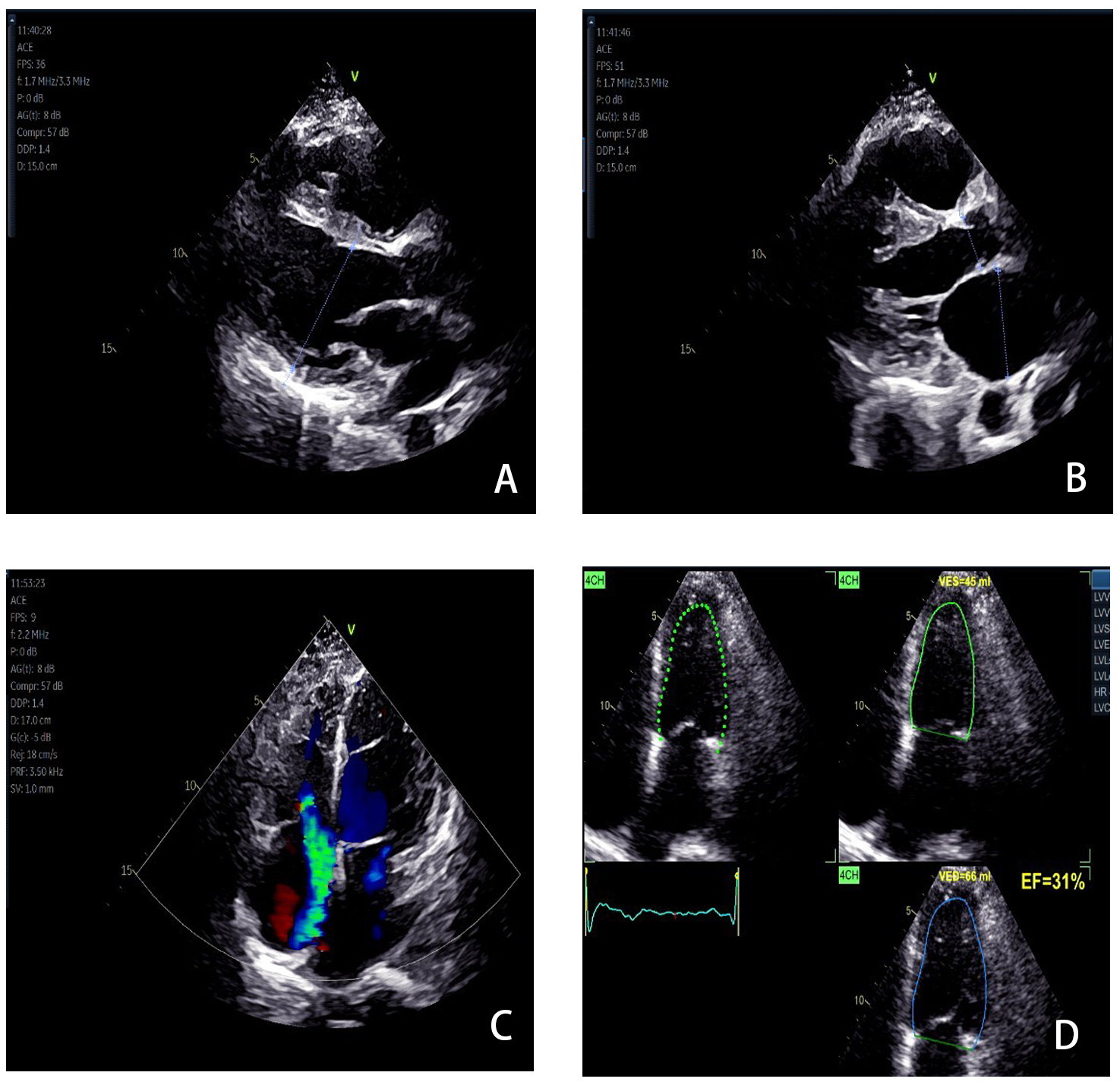


**Supplemental Figure 8.** Echocardiography of patient 3. A-D, the images on left ventricular long axis section, aorta short axis section, four-chamber view, two-chamber view indicating 40 mm of left atrial thickness, 45 mm of right atrial thickness, 51 mm of LVEDD, 11 mm of interventricular septal thickness, 8 mm of LVPT, 25 mm of pulmonary artery width, 38 mmHg of pulmonary arterial pressure and 33% of LVEF.


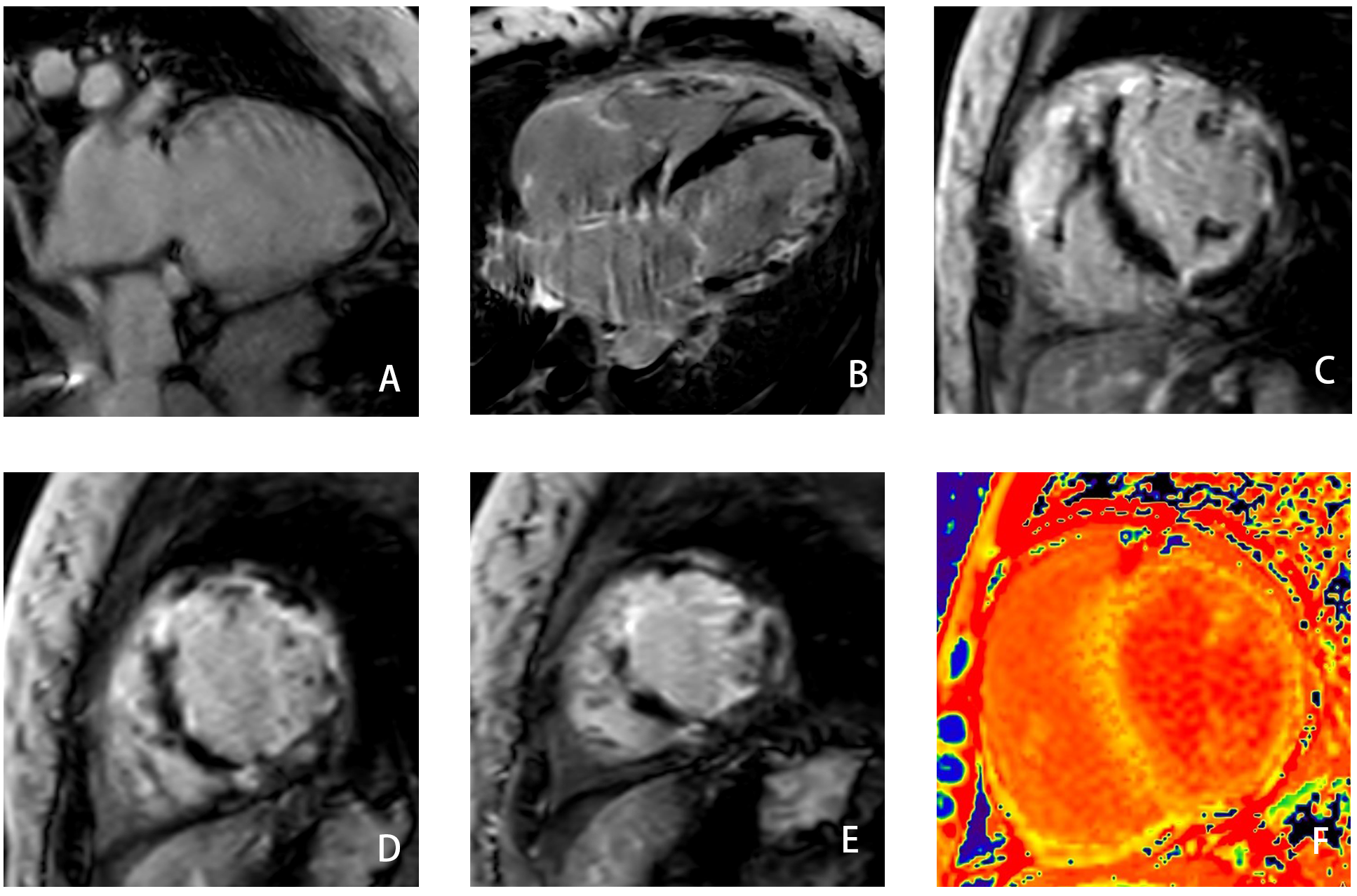


**Supplemental Figure 9.** Cardiac cine-MRI images of patient 3. A, the two-chamber long axis images of heart showing normal LVWT, 27% of LVEF and formation of thrombus at apex (arrow). B-E, four-chamber long axis images and short axis PSIR delayed enhancement on base, central and apex of LV showing extensive LGE mainly in endocarditis, partly transmural and gradually increased from base to apex. Patchy LGE appearing in septum wall. F, T1 mapping with 1893.3ms of T1.
